# Supplementary material for: Indolylazine Derivative Induces Chaperone Expression in Aged Neural Cells and Prevents the Progression of Alzheimer’s Disease
Source: Molecules. 2022 Dec 15;27(24):8950. doi: 10.3390/molecules27248950 (PMC9785687; doi:10.3390/molecules27248950)
Supplement: Supplementary file 1 [file molecules-27-08950-s001.zip › molecules-2057128-supplementary.pdf]

Supplementary Materials to manuscript

Indolylazine derivative induces chaperone expression in aged neural cells and prevents the progression of Alzheimer's disease

Vladimir F. Lazarev[1], Elizaveta A. Dutysheva[1], Elena R. Mikhaylova[1], Maria A. Trestsova[2], Irina A. Utepova[2], Oleg N. Chupakhin[2], Boris A. Margulis[1], Irina V. Guzhova[1*]

NMR Spectra

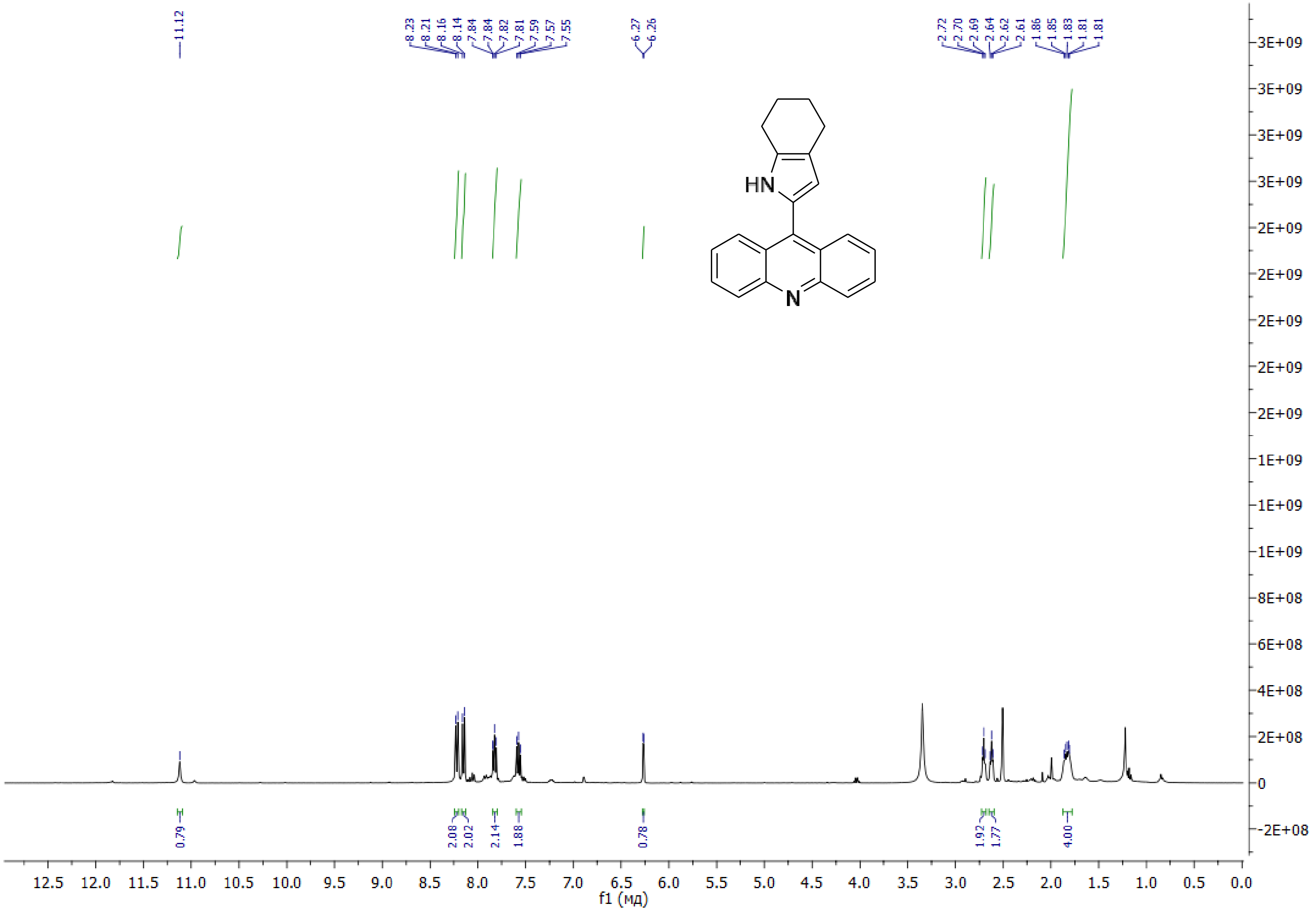

Figure S1. ^{1}H NMR spectrum for 9-(4,5,6,7-tetrahydro-1H-indol-2-yl)acridine (**3**).

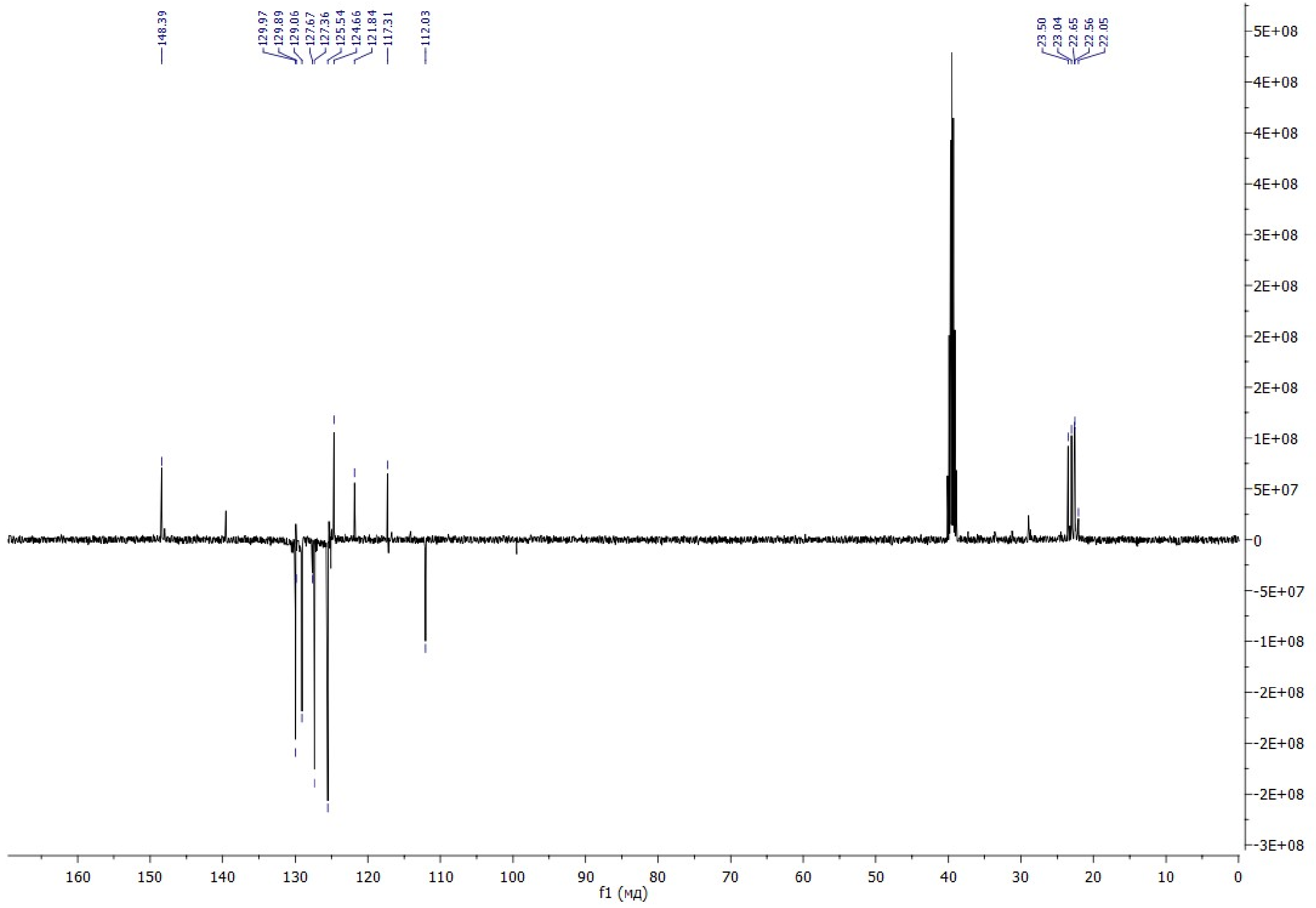

Figure S2. ^{13}C NMR spectrum for 9-(4,5,6,7-tetrahydro-1H-indol-2-yl)acridine (**3**).

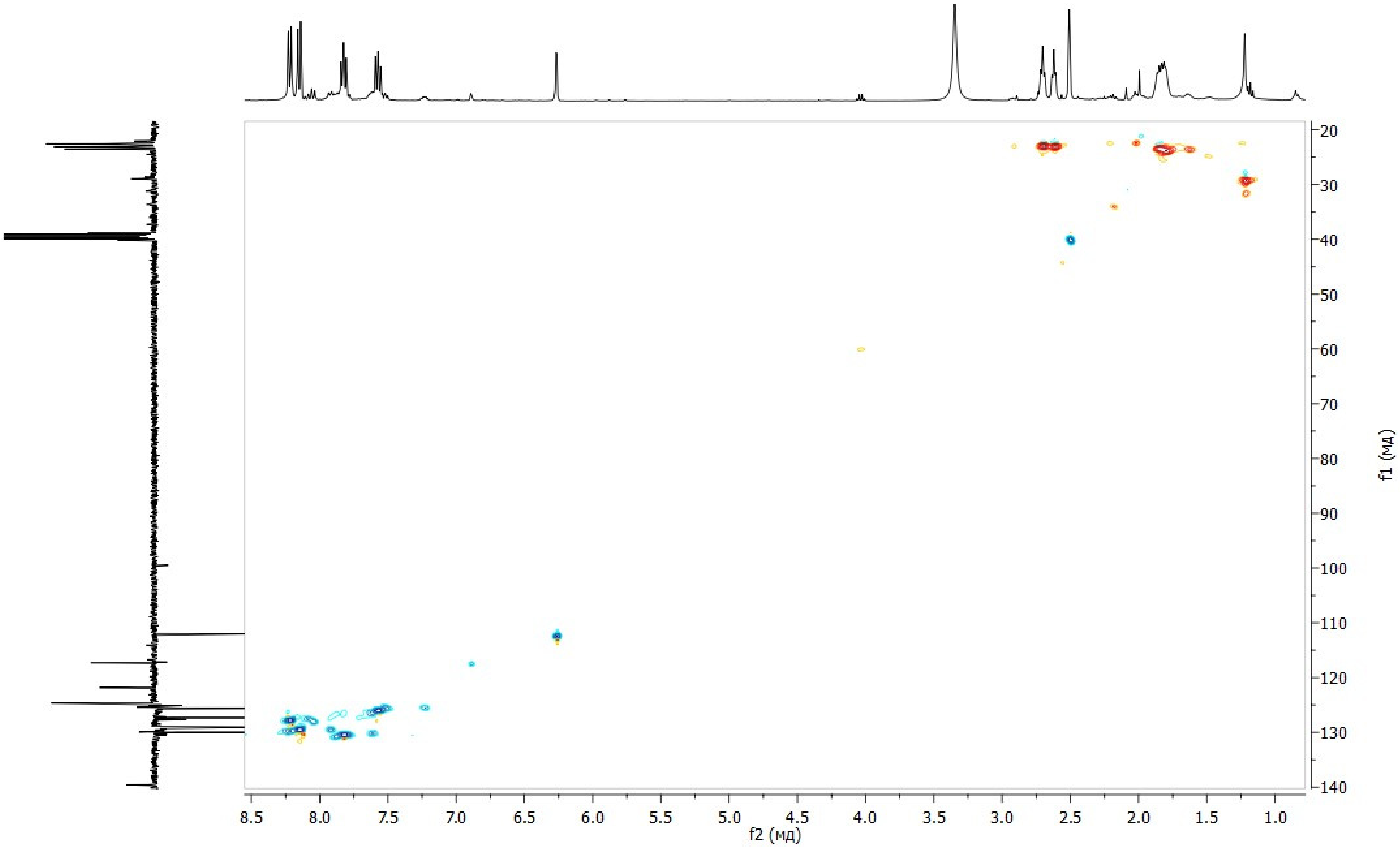

Figure S3. 2D ^{1}H-^{13}C HSQC spectrum for 9-(4,5,6,7-tetrahydro-1H-indol-2-yl)acridine (**3**).

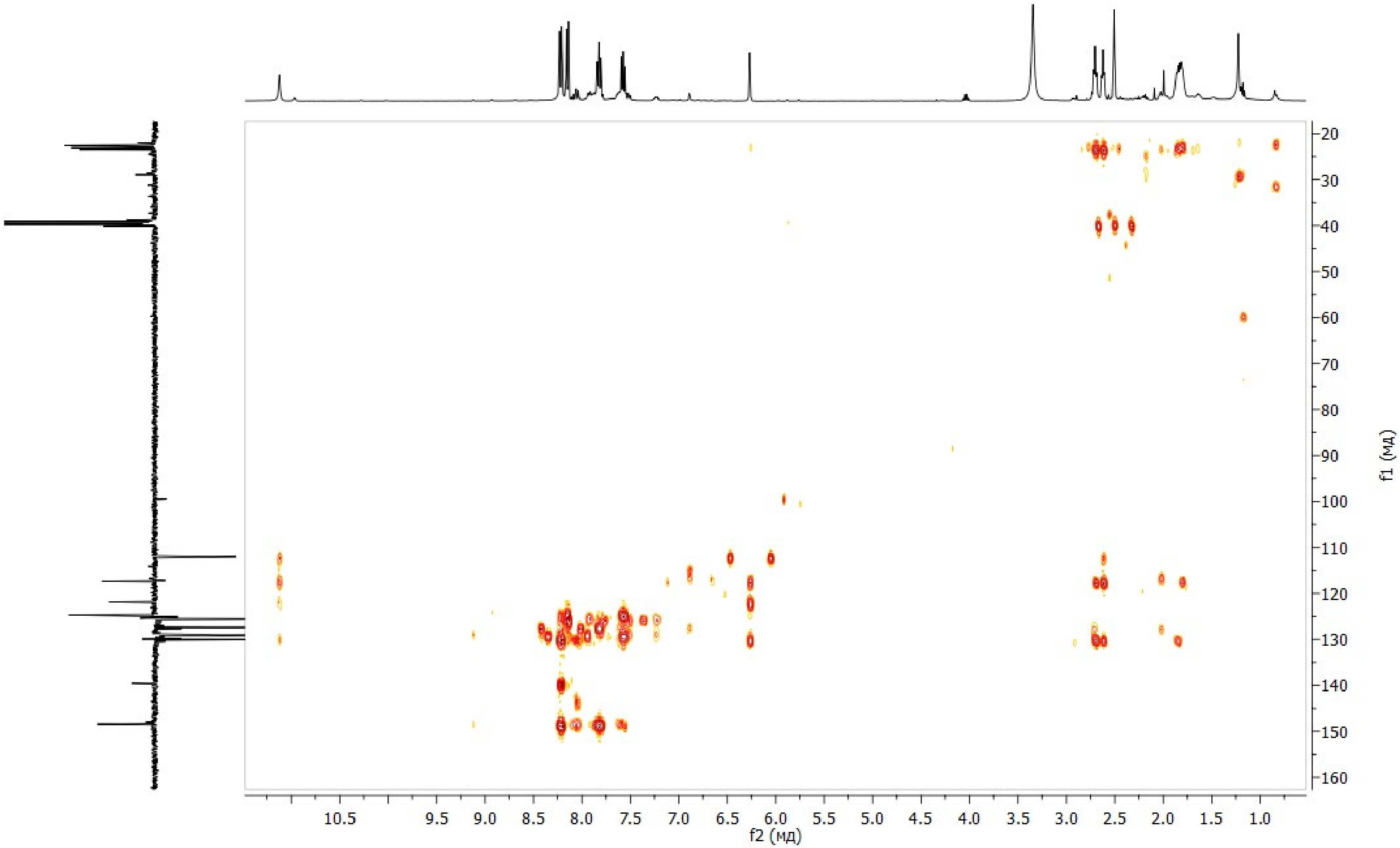

Figure S4. 2D ^{1}H-^{13}C HMBC spectrum for 9-(4,5,6,7-tetrahydro-1H-indol-2-yl)acridine (**3**).

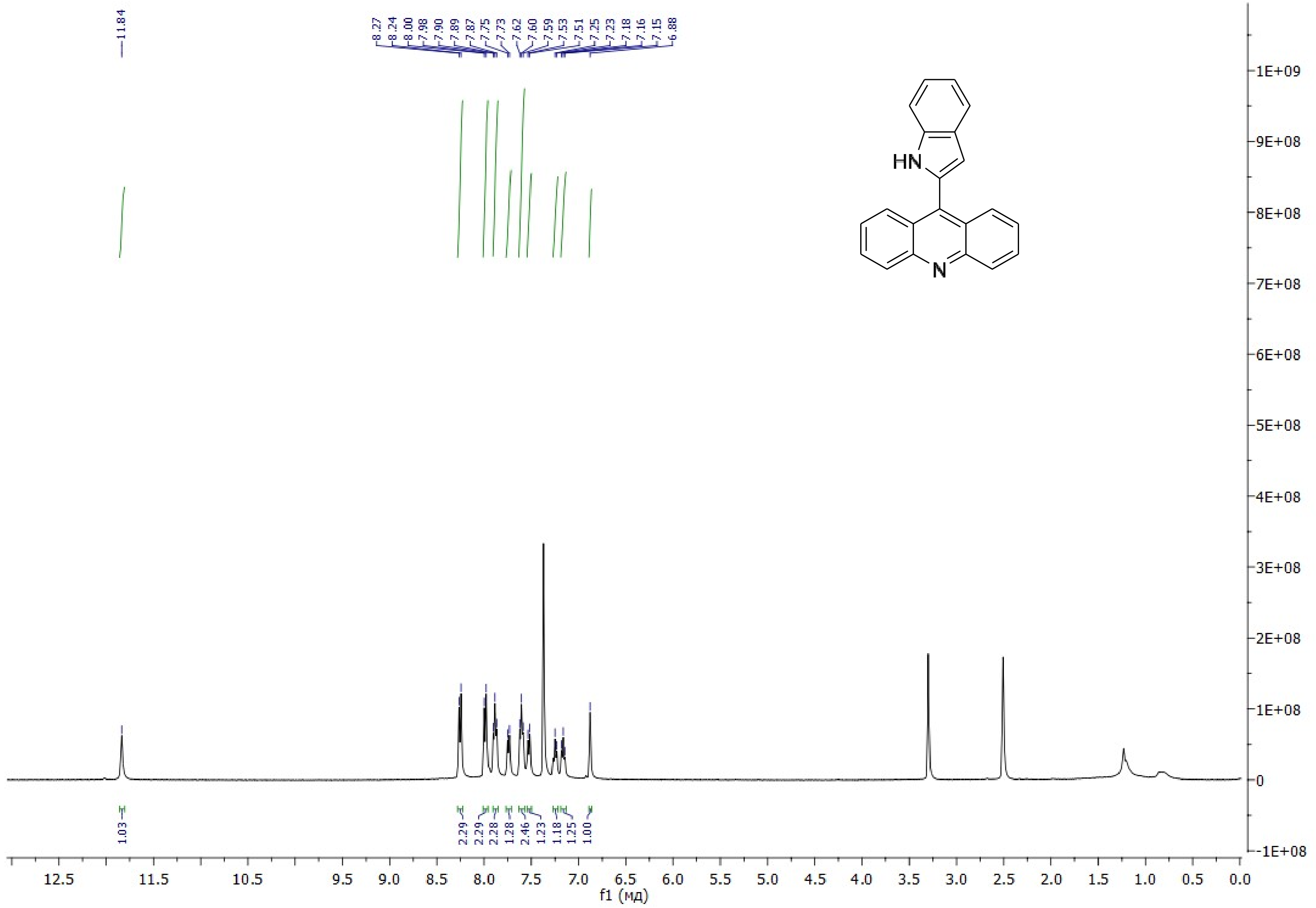

Figure S5. ^{1}H NMR spectrum for 9-(1H-indol-2-yl)acridine (**IA-50**).

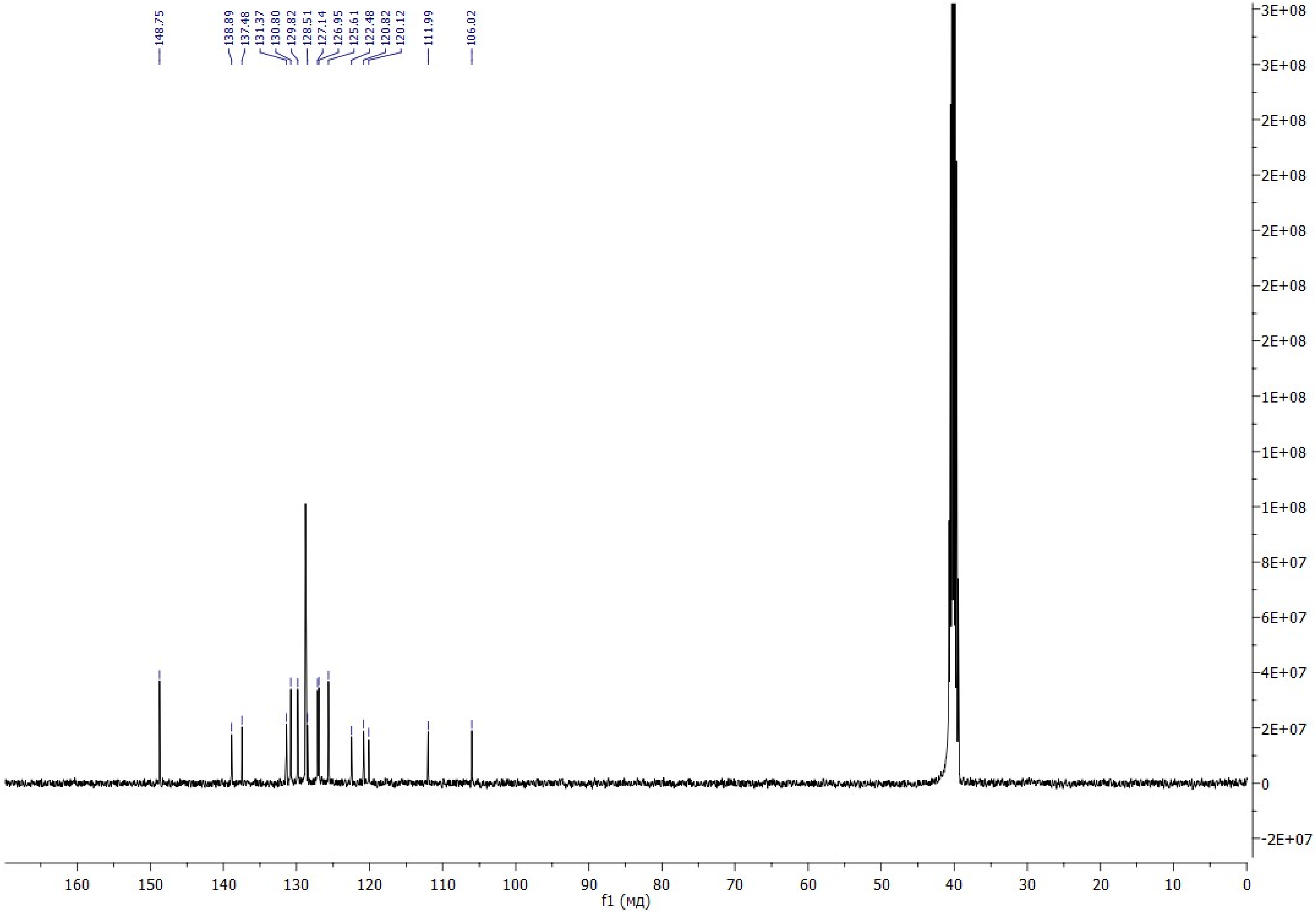

Figure S6. ^{13}C NMR spectrum for 9-(1H-indol-2-yl)acridine (**IA-50**).

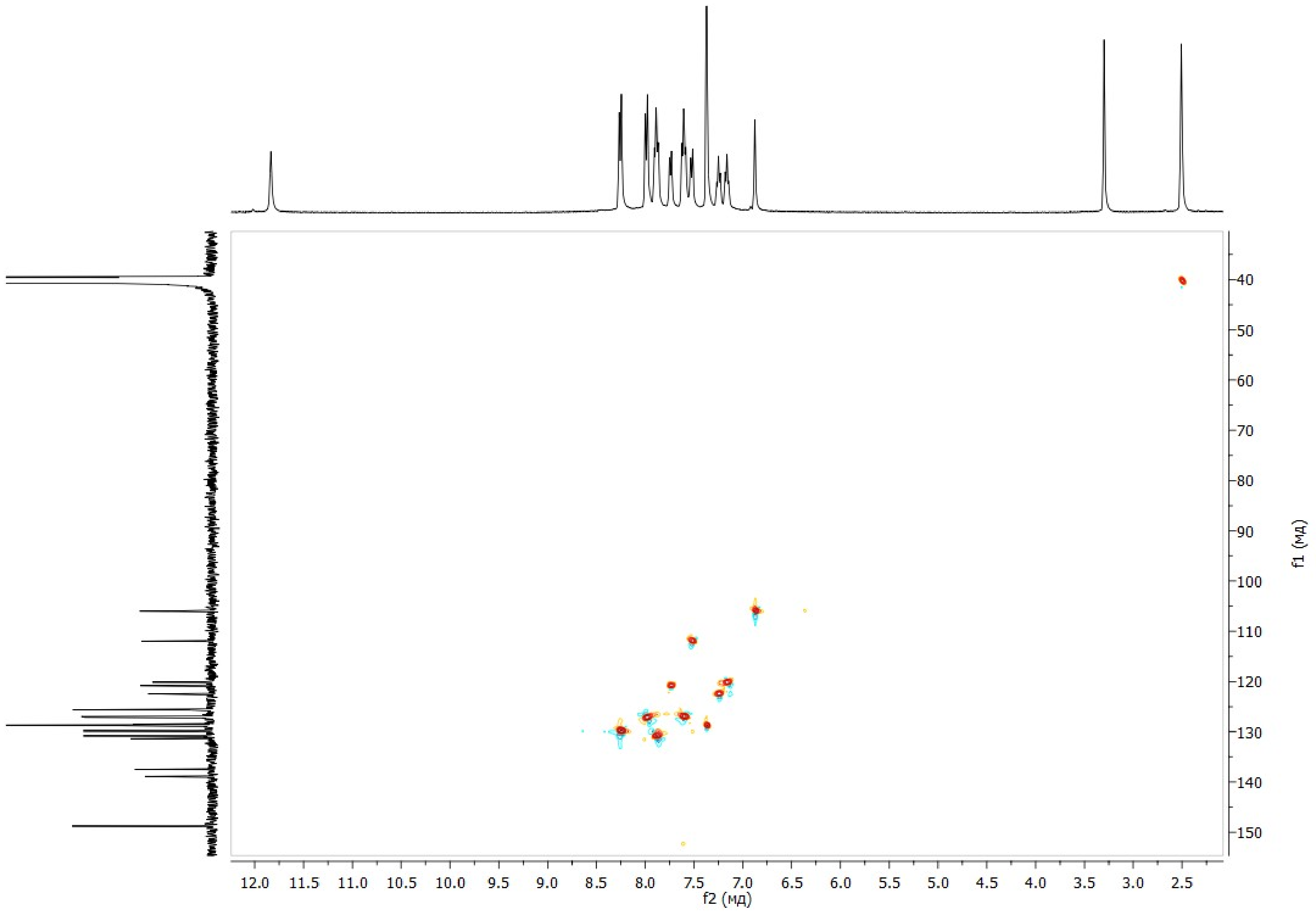

Figure S7. 2D ^{1}H-^{13}C HSQC spectrum for 9-(1H-indol-2-yl)acridine (**IA-50**).

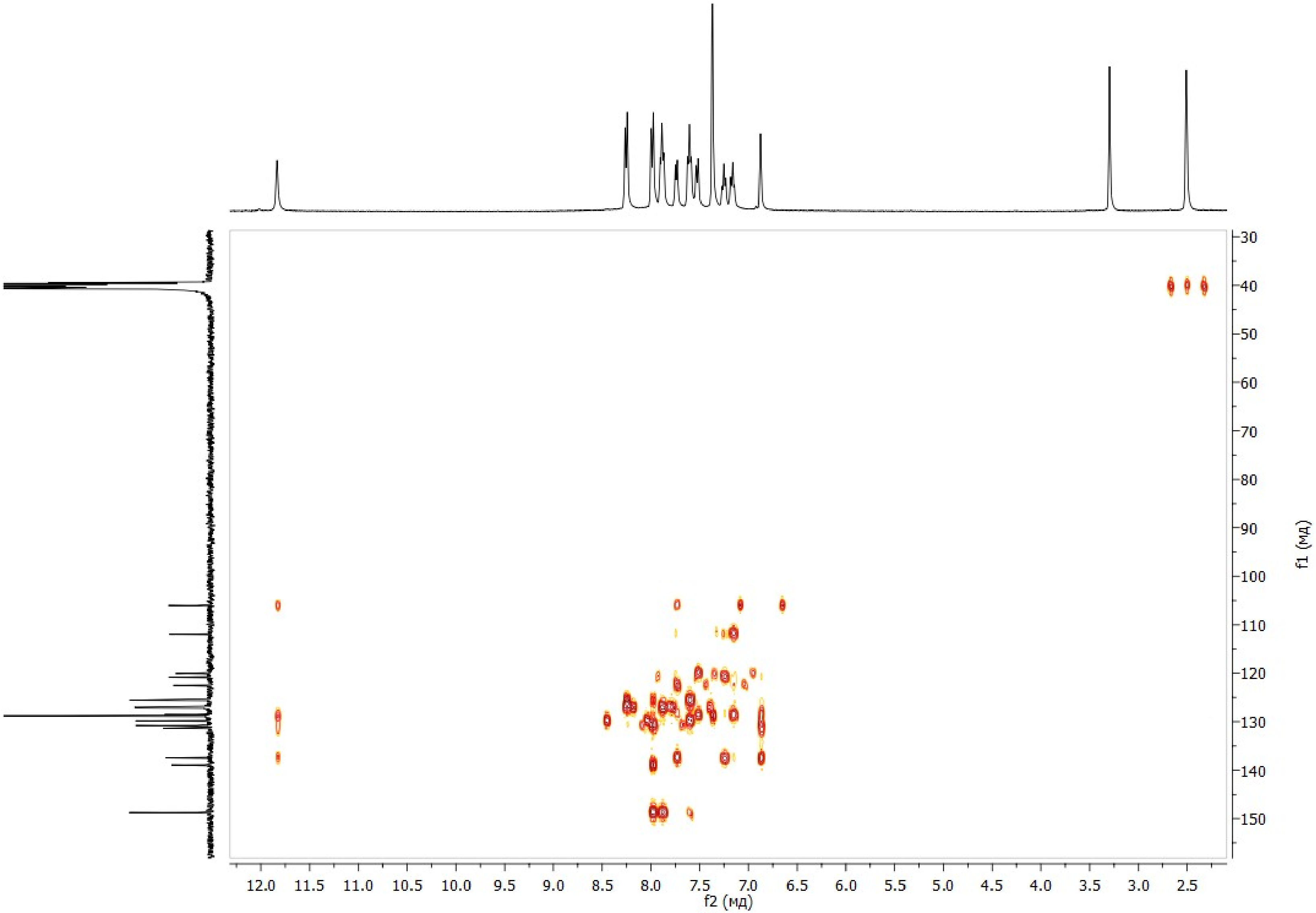

Figure S8. 2D ^{1}H-^{13}C HMBC spectrum for 9-(1H-indol-2-yl)acridine (**IA-50**).
